# Enhancement of Feed Source through Three Dimensional Printing

**DOI:** 10.3390/mi14061244

**Published:** 2023-06-13

**Authors:** Sujan Shrestha, Syed Muzahir Abbas, Mohsen Asadnia, Karu P. Esselle

**Affiliations:** 1 School of Engineering, Faculty of Science and Engineering, Macquarie University, Sydney, NSW 2109, Australia; syed.abbas@mq.edu.au (S.M.A.); mohsen.asadnia@mq.edu.au (M.A.); karu.esselle@uts.edu.au (K.P.E.); 2 School of Electrical and Data Engineering, University of Technology, Sydney, NSW 2007, Australia; 3 Ericsson Australia Pty Ltd., Sydney, NSW 2113, Australia

**Keywords:** 3D printed prototype, radiation patterns, microwave communication

## Abstract

The three-dimensional printed wideband prototype (WBP) was proposed, which is able to enhance the horn feed source by generating a more uniform phase distribution that is obtained after correcting aperture phase values. The noted phase variation obtained without the WBP was $163.65^{\circ }$ for the horn source only, which was decreased to $19.68^{\circ }$, obtained after the placement of the WBP at a λ/2 distance above the feed horn aperture. The corrected phase value was observed at 6.25 mm (0.25λ) above the top face of the WBP. The use of a five-layer cubic structure is able to generate the proposed WBP with dimensions of 105 mm × 105 mm × 37.5 mm (4.2λ× 4.2λ× 1.5λ), which can improve directivity and gain by 2.5 dB throughout the operating frequency range with a lower side lobe level. The overall dimension of the 3D printed horn was 98.5 mm × 75.6 mm × 192.6 mm (3.94λ× 3.02λ× 7.71λ), where the 100 % infill value was maintained. The horn was painted with a double layer of copper throughout its surface. In a design frequency of 12 GHz, the computed directivity, gain, side lobe level in H- and E- planes were 20.5 dB, 20.5 dB, −26.5 dB, and −12.4 dB with only a 3D printed horn case and, with the proposed prototype placed above this feed source, these values improved to 22.1 dB, 21.9 dB, −15.5 dB, and −17.5 dB, respectively. The realized WBP was 294 g and the overall system was 448 g in weight, which signifies a light weight condition. The measured return loss values were less than 2, which supports that the WBP has matching behavior over the operating frequency range.

## 1. Introduction

At microwave frequency, a horn antenna has wide applications, and is used as a feed element in large antennas, radar communication, and mobile and satellite communication, as a standard antenna to compare it with other antennas [1]. The modern wireless system requires greater bandwidth with efficient data transmission. This requires the system to maintain a greater frequency band with improved channel capacity and wider bandwidth [2,3]. The gain level of a horn antenna is usually maintained by aperture size and its length. However, higher aperture size results in increased length of the horn. Several studies have been conducted to address this issue, such as structure design across the interior E-plane wall of a pyramidal horn antenna, which is able to decrease side lobe levels and increase the gain of the horn antenna [4]. The growing use of the three-dimensional printing process is used to realize 3D printed horn prototype either through a metal 3D printing process [5,6] or by using a spray of metal on 3D printed parts [7,8]. This has resulted in an increase of costs during fabrication. Additionally, studies on meta surfaces are summarized in [9], which shows the flexible capability of microwave metamaterials in the manipulation of electromagnetic waves. It has been widely used in antenna design in recent years due to its planar structure and its potential to enhance antenna performance in terms of directivity, gain, and side lobe levels. The printed patches are generally used to realize the subwavelength meta surfaces as studied in [10], maintaining a proper thickness of dielectric plate and gap between nearby patches [11], a series of subwavelength metallic split ring resonators [12], use of multilayer non uniform meta surfaces that are formed by squares and ring metal patches [13], and an ultrathin metasurfaces lens developed to provide transmission phase compensation for a wide flare angle conical horn [14]. However, these metasurfaces have limited adjusting capacity to electromagnetic waves so difficulties arise in the further application of antenna design. In addition, a metal wideband phase correcting structure was proposed in [15] but it shows a higher value in aperture phase distribution and the required defined distance between two metal layers showing a complex geometrical structure. Interestingly, the application of 3D printing is widely used in the microwave field [16]. A dielectric loaded profile conical horn antenna was designed in [17], which uses more dielectric materials to be filled. In order to have a low profile, a light weight and the use of fewer 3D printed materials with a lower side lobe level, a cube structure was considered, as in [18], to show a reduced side lobe level, whereas in [19] the considered structure was able to maintain phase correction for about $40^{\circ }$. The advancement in additive manufacturing technologies is growing in significance in the field of electromagnetic wave propagation; these were studied in [20,21,22,23,24]. Considering this aspect, the novelty of this manuscript was in proposing a simple, low profile, easily realizable geometrical structure that will be able to have a more uniform phase distribution, which can be further analyzed to study the deviation of the radiated beam direction. Hence, we designed a low profile, light weight, 3D printed horn antenna through Fused Deposition Modeling (FDM) with a double layer of copper throughout its surface, which is fed by a WR-75 waveguide through the bottom part. The five layers of the cube structure generated by the Multijet Printing technique was able to generate a more uniform phase distribution that shall ultimately improve the performance of the feed source. The proposed work shows efficiency in the utilization of the 3D printing technique in realizing the meta surface. The proposed structure, which was realized by a defined cube pattern, and size was helpful for understanding phase uniformity analysis against other literature studies performed in [13,14,15,17]. This article is organized as follows: Section 1 describes the requirements for the WBP structure. Similarly, Section 2 depicts the generation of a five-layer cube structure and its proper placement in the defined aperture position. Section 3 presents the corrected phase values observed in the operating frequency band. Section 4 highlights the obtained simulated and experimental results along with the uniqueness of the proposed WBP. Section 5 presents the conclusions.

## 2. Generation of Wide Band Prototype

**Figure 1:**
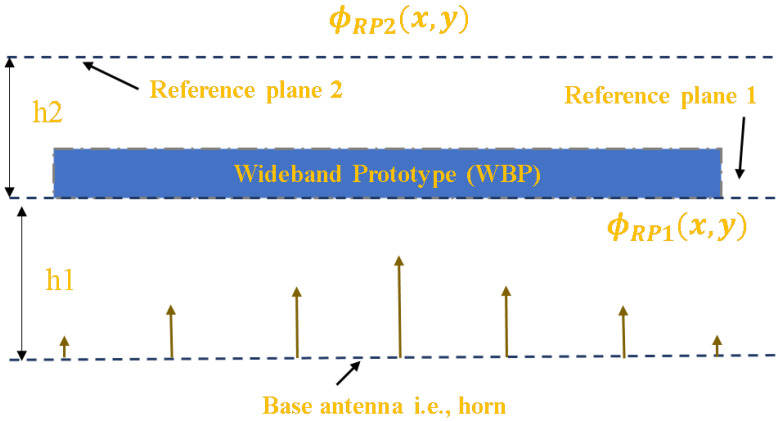
System overview with proposed WBP.

The realized WBP was placed at a defined h1 = 12.5 mm (λ/2) distance from the base antenna as shown in Figure 1. We considered an aperture dimension of the horn of 4λ × 4λ (λ = 25 mm at 12 GHz). The variation in the phase of $163.65^{\circ }$ was noted above the feed horn source, which was decreased to $19.68^{\circ }$ after the placement of the WBP at h2 = 6.25 mm (λ/4) above the top face of the WBP.

**Figure 2:**
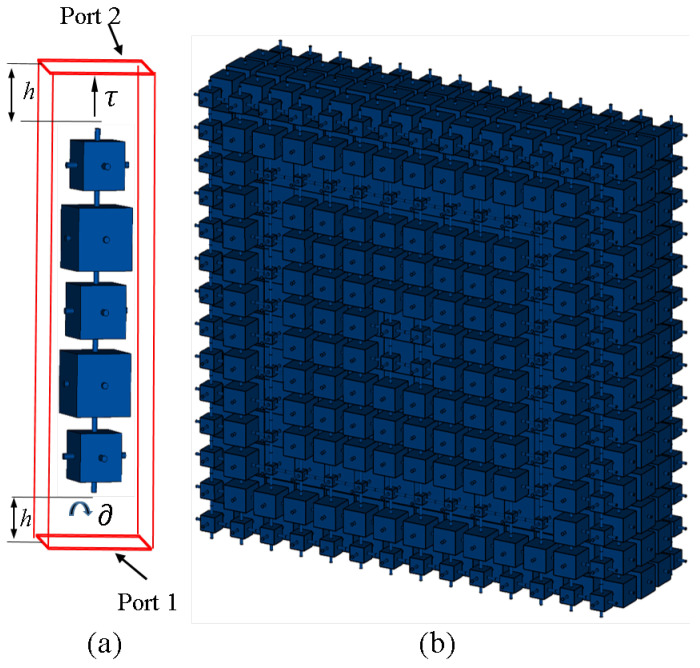
(**a**) Building block. (**b**) Perspective glimpse of WBP.

**Figure 3:**
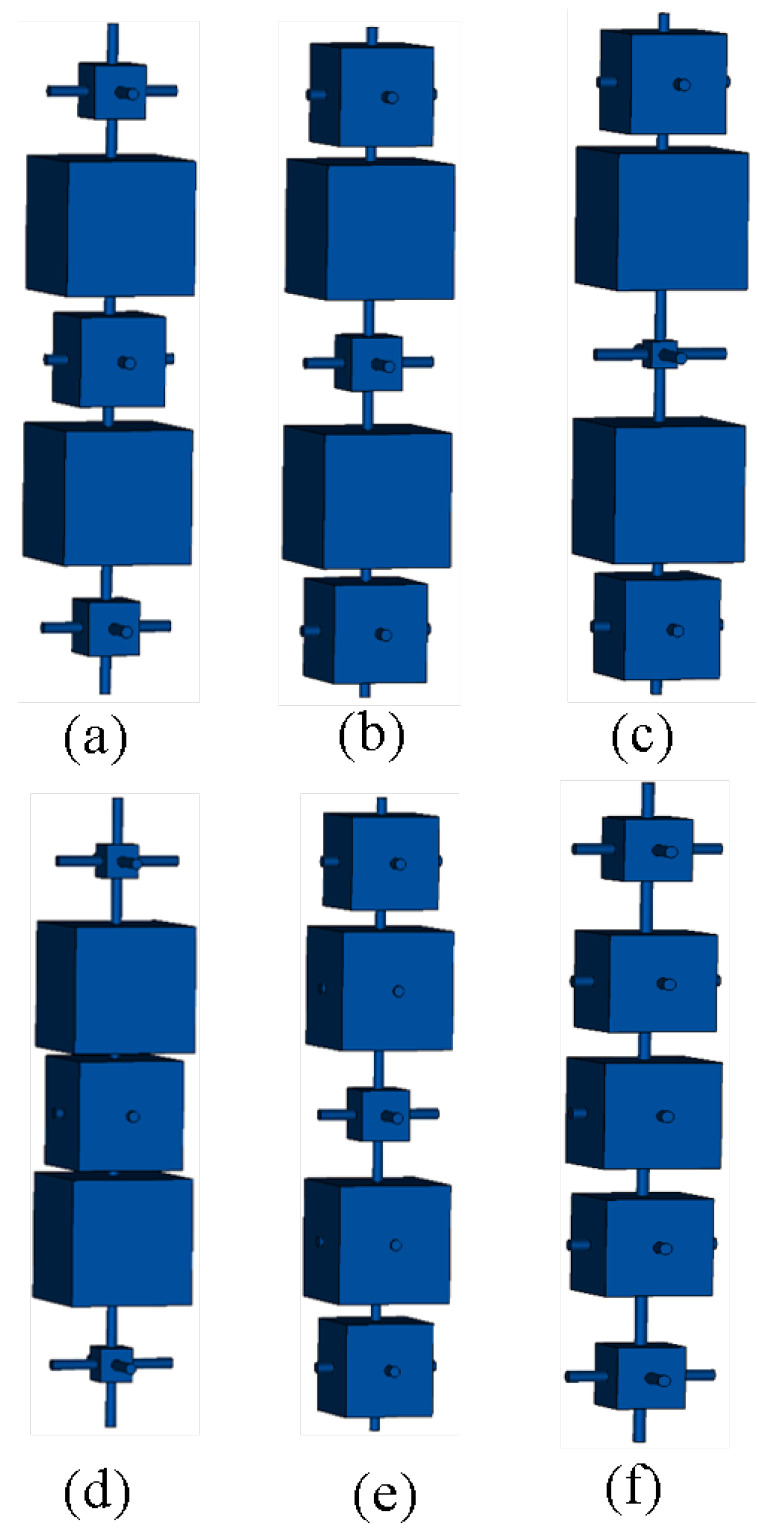
Building block of cubes in (**a**) Round 1 (**b**) Round 2 (**c**) Rounds 3 and 4 (**d**) Round 5 (**e**) Round 6 (**f**) Round 7.

The proposed WBP prototype, along with the unit cell arrangement, are shown in Figure 2. The cubes arranged in five layers are shown in Figure 2a, where the variation in cube dimension in each respective layer generates a corresponding change in the transmission magnitude and phase values. We set the cubes’ dimensions as x1, x2, x3, x2, x1, and arranged them from Port 2 to Port 1. The variation in cube size was from a minimum of 0.5 mm (0.02λ) to a maximum of 7.5 mm (0.3λ). The proposed WBP perspective view is depicted in Figure 2b. The various cube dimensions, as arranged in five layers, are shown in Figure 3. The respective rounds were maintained from a central position of WBP across the end aperture dimension, where Round 1 lies in a central position, whereas Round 7 appears towards the end portion. The higher transmission magnitude (|S21| > 0.8) was maintained to provide an enhanced gain improvement. Round 1, Round 2, Round 3, Round 4, Round 5, Round 6, and Round 7 varied from 3.75 mm, 11.25 mm, 18.75 mm, 26.25 mm, 33.75 mm, 41.25 mm, to 48.75 mm across the aperture dimension. Overall, we had 196 unit cells that were accompanied in the WBP structure.

The relative dimensions of the cubes were noted from normalized phase values that were calculated in the h1 position. Synthesis algorithms prepared for generation of proposed prototype are highlighted in the below steps.

Step 1: Calculate the required phase value at a 0.5λ distance above the defined aperture of the horn feed source. Phase values obtained in aperture positions of 3.75 mm, 11.25 mm, 18.75 mm, 26.25 mm, 33.75 mm, 41.25 mm, and 48.75 mm from the center of the aperture are $61.98^{\circ }$, $51.16^{\circ }$, $44.13^{\circ }$, $44.51^{\circ }$, $38.92^{\circ }$, $8.53^{\circ }$, and $-55.92^{\circ }$.
Step 2: Normalize these noted phase values by considering higher phase values above the highest value. Here, we considered $240^{\circ }$ and the normalized values obtained were $180^{\circ }$, $190^{\circ }$, $195^{\circ }$, $195^{\circ }$, $200^{\circ }$, $230^{\circ }$, and $295^{\circ }$ for defined aperture positions of 3.75 mm, 11.25 mm, 18.75 mm, 26.25 mm, 33.75 mm, 41.25 mm, and 48.75 mm.
Step 3: These normalized phase values are correlated to respective cube sizes from a database prepared with an arrangement of five layers of cubes. The respective cube sizes are 3, 7.5, 7.5 mm${}^{3}$ in 3.75 mm; 1.5, 7.5, 7.5 mm${}^{3}$ in 11.25 mm; 3.5, 7.5, 7 mm${}^{3}$ in 18.75; 3.5, 7.5, 7 mm${}^{3}$ in 26.25 mm; 2.5, 7.5, 7 mm${}^{3}$ in 33.75 mm; 1.5, 7.5, 6 mm${}^{3}$ in 41.25 mm; and 1.5, 7, 2.5 mm${}^{3}$ in 48.75 mm.
Step 4: Furthermore, cube sizes are tuned to appropriate values to maintain uniformity in the phase distribution at 6.25 mm (λ/4) above the top face of the WBP, which is based on the prepared five-layer cubes database. Thus, the obtained respective cube sizes are 3, 7.5, 5 mm${}^{3}$ in 3.75 mm; 5.5, 7.5, 3 mm${}^{3}$ in 11.25 mm; 5.5, 7.5, 1.5 mm${}^{3}$ in 18.75; 5.5, 7.5, 1.5 mm${}^{3}$ in 26.25 mm; 2, 7.5, 6.5 mm${}^{3}$ in 33.75 mm; 5.5, 7, 3 mm${}^{3}$ in 41.25 mm; and 3.5, 5.5, 6 mm${}^{3}$ in 48.75 mm. These cubes are arranged for seven different rounds as Round 1, Round 2, Round 3, Round 4, Round 5, Round 6, and Round 7.

The WBP is suitable to manufacture using 3D printing techniques that utilize Vero CMYK ($\epsilon_{r}$ = 2.8 and tanδ 0.124). The respective size of the cylindrical rods and cube dimension variation are detailed in [19]. The length of perpendicular cylinders is maintained at 7.5 mm (0.3λ), which holds the cubes of defined dimensions. The cubes’ dimension is increased in a step size of 0.5 mm (0.02λ) from minimum dimensions of 0.5 mm${}^{3}$ until the maximum dimension of 7.5 mm${}^{3}$. The optimal value of the cube size is 7.5 mm${}^{3}$ and analytical calculations are performed to obtain the respective values of transmission magnitude and phase variations. The generated transmission magnitude and phase are arranged for specific layers of cubes in a five-layer structure. The phase values that correspond to the transmission magnitude of unity are considered and arranged in tabular form. The generated transmission magnitude and phase values for the arrangement of five layers of cubes are detailed in Table 1 and Table 2.

## 3. Phase Correction as Observed above WBP

**Figure 4:**
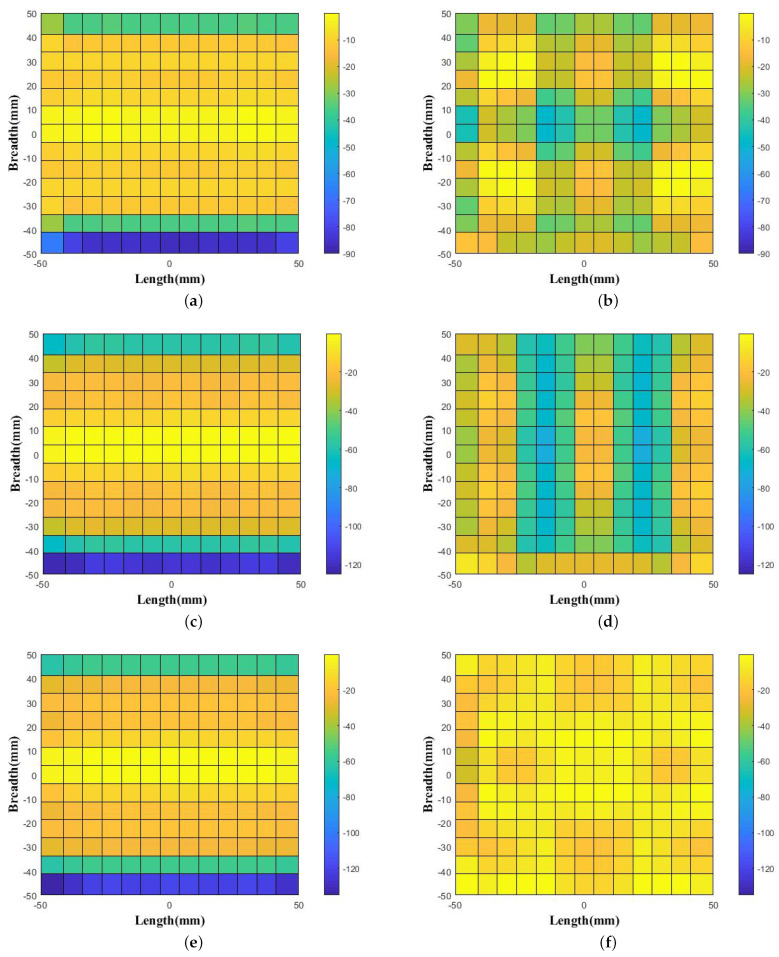
Aperture Phase values observed above the feed horn without WBP (**a**) at 10 GHz, (**c**) at 11 GHz, and (**e**) at 12 GHz and with the placement of WBP (**b**) at 10 GHz, (**d**) at 11 GHz, and (**f**) at 12 GHz.

**Figure 5:**
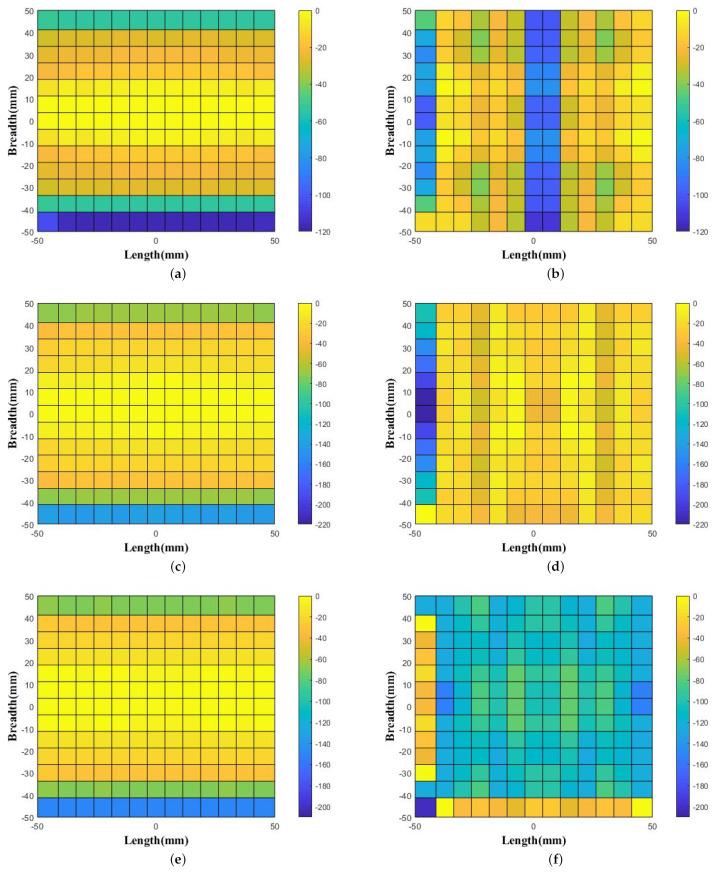
Aperture Phase values observed above feed horn without WBP (**a**) at 13 GHz, (**c**) at 14 GHz, and (**e**) at 15 GHz and with the placement of WBP (**b**) at 13 GHz, (**d**) at 14 GHz, and (**f**) at 15 GHz.

The generated uniform distribution of phase patterns were observed as shown in Figure 4 and Figure 5. As noticed, for the horn source-only case, the phase variation is $86^{\circ }$ in 10 GHz, $122^{\circ }$ in 11 GHz, $132^{\circ }$ in 12 GHz, $117^{\circ }$ in 13 GHz, $143^{\circ }$ in 14 GHz, and $151^{\circ }$ in 15 GHz. Interestingly, these phase variations are decreased with the effect of WBP placement above the feed source. The noted uniform phase variations are $32^{\circ }$ in 10 GHz, $41^{\circ }$ in 11 GHz, $19^{\circ }$ in 12 GHz, $111^{\circ }$ in 13 GHz, $48^{\circ }$ in 14 GHz, and $117^{\circ }$ in 15 GHz.

**Figure 6:**
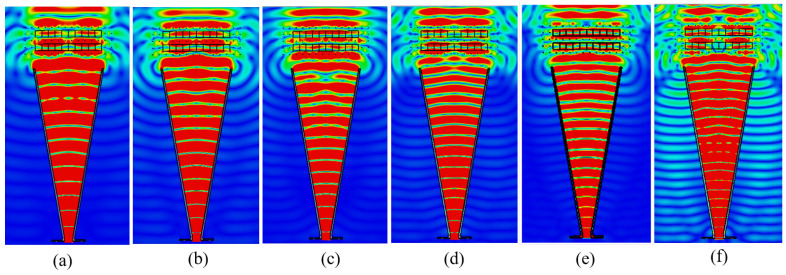
Planar wave fronts observed above WBP (**a**) 10 GHz (**b**) 11 GHz (**c**) 12 GHz (**d**) 13 GHz (**e**) 14 GHz (**f**) 15 GHz.

Moreover, uniformity in the conversion of spherical to planer wave-fronts were observed as highlighted in Figure 6.

## 4. Result and Discussion

We used CST-Microwave Studio to calculate the various performance criteria of the WBP structure. The noted VSWR is less than 2 from 10 GHz to 15 GHz of the operating frequency band that signifies wideband operation.

**Figure 7:**
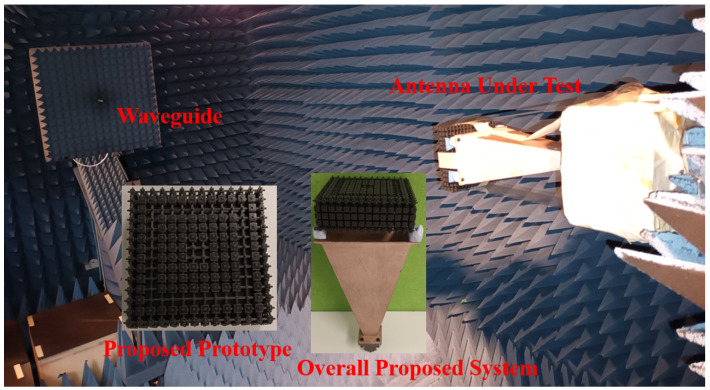
Measurement system showing the experimental setup of proposed wideband prototype.

The experimental setup was carried out in an NSI-700S-50 spherical near field measurement system at the Australian Antenna Measurement Facility, which is shown in Figure 7. The figures attached show the fabricated WBP structure and the overall assembled system with the feed source. Further sections detail radiation patterns and characteristic plots.

**Figure 8:**
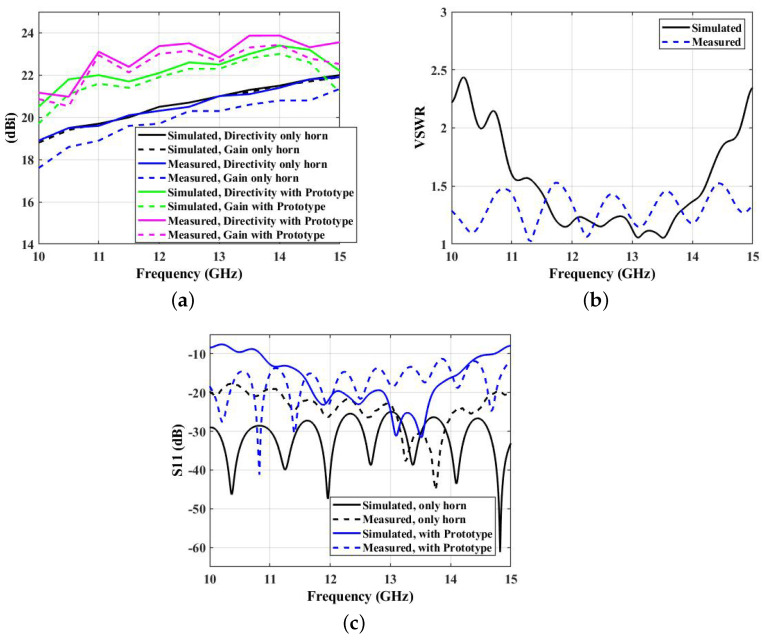
(**a**) Simulated and measured broadside directivity and gain plots. (**b**) Simulated and measured VSWR over 10 to 15 GHz frequency range. (**c**) Simulated and measured S11 parameter over 10 to 15 GHz frequency range.

Figure 8a signifies the performance matrices of the overall system with and without WBP structure. The overall improvement of 2.5 dBi in broadside directivity and gain were noted throughout the operating frequency range. This highlights the fact that the performance of the horn can be enhanced by compromising its aperture dimension and with the use of the proposed 3D printed WBP structure. The voltage standing wave ratio (VSWR) is shown in Figure 8b, depicting the wideband performance of the overall system. VSWR values are less than 2.2 over the operating frequency range. Measured VSWR depicts the matching condition of the proposed WBP structure. Similarly, the S11 plot, as shown in Figure 8c, shows the wideband characteristics of the proposed system where S11 values are less than −10 dB over the operating frequency band from 10 to 15 GHz. The simulated and measured values with and without the proposed prototype show better S11 values in the Ku-band of the operating frequency range.

Table 3 shows an improvement in measured directivity and gain values after the placement of the proposed WBP structure above the feed source. Those values are compared against the simulated results. As noticed, in a design frequency of 12 GHz, simulated directivity, gain, and measured directivity, gain for the horn-only case are 20.489, 20.463, 20.310, 20.335 dBi, which are improved by around 2.5 dBi resulting in a simulated directivity, gain and measured directivity, and gain with the proposed prototype of 22.1, 21.9, 23.372, and 23.007 dBi, respectively. Throughout the operating frequency band, the overall performances in the directivity and gain margins are improved.

**Figure 9:**
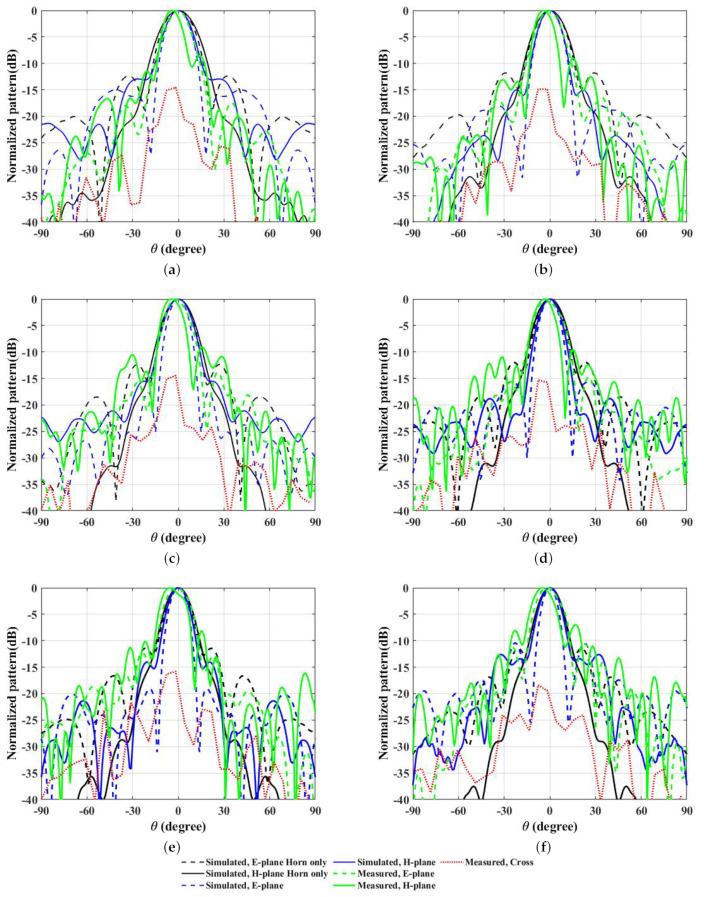
Simulated and measured radiation patterns of overall system with WBP structure above the feed source are shown in Figure 9 (**a**–**f**) for 10 GHz, 11, GHz, 12 GHz, 13 GHz, 14 GHz, and 15 GHz frequencies.

The observed radiation patterns of the overall system are depicted in Figure 9, highlighting the narrow beamwidth and lower side lobe levels in 10, 11, 12, 13, 14 and 15 GHz frequencies as noted in E- and H- planes, respectively. In E- and H- planes, the noted side lobe levels are −12.4, −11.8, −12.4, −12, −11.7, −11.4 dB and −27.7, −29.9, −26.5, −26.5, −25.6, −29.1 dB with the horn-only case, as compared against −15, −18, −17.5, −15.7, −19.1, −10.4 dB and −12.9, −15, −15.5, −18.8, −14, −12.6 dB with the proposed WBP surface placed above the horn feed source. In the condition with the horn-only case, in the H-plane of frequencies 10, 11, 12, 13, 14, and 15 GHz simulated directivities are 18.9, 19.7, 20.5, 21, 21.5 and 22 dBi; simulated gains are 18.8, 19.7, 20.5, 21, 21.5 and 21.9 dBi; 3 dB angular widths are $21.4^{\circ }$, $19.2^{\circ }$, $17.6^{\circ }$, $16.5^{\circ }$, $15.4^{\circ }$ and $14.6^{\circ }$; side lobe levels are −25.8, −30.7, −31.1, −26.5, −27.6 and −29.1 dB. Similarly, in the E-plane of frequencies, the 3 dB angular widths are $20.1^{\circ }$, $18.5^{\circ }$, $16.5^{\circ }$, $15.4^{\circ }$, $14.5^{\circ }$, and $13.6^{\circ }$; side lobe levels are −12.2, −12.4, −12, −12, −11.7, and −11.4 dB over the operating frequency range. Additionally, an improvement in the characteristics plots was observed with the WBP structure placed above the feed horn source. In the condition with the WBP and horn source, for the H-plane of frequencies 10, 11, 12, 13, 14, and 15 GHz simulated directivities and gains are 20.5, 22, 22.1, 22.5, 23.4 and 22.2 dBi; 19.7, 21.6, 21.9, 22.3, 23 and 21.2 dBi; 3 dB angular widths are $16.2^{\circ }$, $14.3^{\circ }$, $15.8^{\circ }$, $13.4^{\circ }$, $12.9^{\circ }$, and $13^{\circ }$; side lobe levels are −12.9, −15, −15.5, −18.8, −14, and −12.6 dB. In E-plane of frequencies 10, 11, 12, 13, 14 and 15 GHz, 3 dB Angular width are $14.9^{\circ }$, $13.9^{\circ }$, $12.3^{\circ }$, $11.8^{\circ }$, $10.8^{\circ }$, and $9.8^{\circ }$; side lobe levels are −15, −18, −17.5, −15.7, −19.1, and −10.4 dB. Interestingly, measured directivity and gain values in 10, 11, 12, 13, 14, 15 GHz frequencies are 21.2, 23.2, 23.4, 22.9, 23.9, 23.6 dBi and 20.9, 22.9, 23.1, 22.7, 23.5, 22.6 dBi, respectively. The standard horn antenna [25] with an overall height of around 225 mm was considered to realize the analysis of aperture phase uniformity, which is supportive of understanding the requirement of the full phase of $360^{\circ }$. This will be helpful for understanding the metasurface required for the beam steering and beam deviation phenomena. The use of the proposed superstrate is applicable for standard gain horn antenna with a defined length. The shorter horn with a lower gain was unable to depict the beam steering or deviation phenomena, even though the superstrate enhances the gain. This is due to the aperture phase correction requirement where a standard gain horn antenna with a defined aperture dimension of 98.5 mm × 75.6 mm is able to generate more uniformity in phase distribution with a lower side lobe level of around −17.5 dB. Table 4 shows the preference of the proposed WBP structure as compared with other design aspects. It shows wide band operation in the Ku-band, ranging from 10 GHz to 15 GHz. The aperture dimension is relatively smaller and has higher directivity and gain values with an improved 3dB bandwidth. Additionally, side lobe levels are comparatively lower, which has generated better radiation patterns.

## 5. Conclusions

This manuscript proposed a 3D printable WBP structure which improves the broadside directivity and gain of the feed source. The proposed five-layer surface was numerically studied through CST-MWS and is able to generate a more uniform phase distribution for the 10 to 15 GHz frequency range. In design frequency, lower side lobe levels of −17.5 dB and −15.5 dB, respectively, in E- and H- planes were obtained. Moreover, an improvement of 2.5 dBi in broadside directivity and gain was observed with better radiation patterns. The overall system was 448 g in weight and the fabricated WBP was 294 g. This signifies the light weight condition of the overall system. The measured return loss was less than 2.2, which shows matching behavior over the operational frequency band. Possible future work could include approaches that will be implemented to realize a 3D-printable beam-steerable surface. This work shows the full phase requirement of $360^{\circ }$, which can be further extended to be implemented in the beam deviation phenomenon. There is no use of dielectrics, which reduces the cost and weight of the overall system. The next possible applications and challenges could be observed in high power systems and radiation patterns synthesis in near field regions. This research has great potential for radar and wireless communication systems due to its capability for greater bandwidth and improved electromagnetic waves transmission. 

## Figures and Tables

**Table 1:** Arrangement of transmission magnitude and phase values with five layer cubes.

Transmission Magnitude	Transmission Phase (Degree)	x1	x2	x3
0.9862	0	5	2	1
0.9951	5	5	0.5	1.5
0.9948	10	0.5	4	4.5
0.9918	15	0.5	4	4
0.9931	20	0.5	3.5	4
0.9922	25	0.5	3.5	3.5
0.9914	30	0.5	3	3.5
0.9894	35	0.5	2.5	3.5
0.9911	40	0.5	2.5	1.5
0.9967	45	0.5	0.5	1.5
0.9968	50	1	0.5	1
0.9710	55	7.5	7.5	6
0.9719	60	7.5	7.5	5.5
0.9719	65	7.5	7.5	5.5
0.9603	70	7	7.5	7
0.9574	75	7.5	7.5	4.5
0.9409	80	7.5	7.5	3.5
0.9366	85	7.5	7.5	0.5
0.8854	90	7	7	7.5
0.9217	95	7	7.5	6
0.9549	100	6	7.5	7.5
0.9372	105	7.5	7	5.5
0.9244	110	7	7.5	5
0.9281	115	5.5	7.5	7.5
0.8599	120	6.5	7.5	6
0.9777	125	7	7.5	2.5
0.9855	130	7.5	7	1.5
0.8603	135	6	7.5	6
0.9578	140	4.5	7.5	7.5
0.9133	145	5	7.5	7
0.8376	150	7	6.5	6
0.9717	155	6.5	7.5	2.5
0.9124	160	5.5	7.5	5.5
0.9717	165	1.5	7.5	7.5
0.9757	170	7	7	1.5
0.9374	175	7.5	3.5	7
0.9775	180	7.5	5.5	4
0.9998	185	2	7.5	7

**Table 2:** Arrangement of transmission magnitude and phase values with five layer cubes.

Transmission Magnitude	Transmission Phase (Degree)	x1	x2	x3
0.9723	190	5.5	7.5	3
0.9705	195	5.5	7.5	1.5
0.9810	200	2	7.5	6.5
0.9859	205	6.5	4.5	7.5
0.9486	210	7	6	3.5
0.9781	215	6.5	6	5
0.9936	220	3	7.5	5
0.9992	225	5.5	7	3.5
0.9785	230	5.5	7	3
0.9817	235	6	5.5	6
0.9897	240	1.5	7.5	3.5
0.9825	245	1.5	7.5	2
0.9956	250	4.5	7	3.5
0.9509	255	6	6	4
0.9912	260	3.5	7	4
0.9894	265	3.5	7	3.5
0.9934	270	3.5	5	7.5
0.9823	275	3.5	7	0.5
0.9861	280	3	7	1
0.9737	285	0.5	7	2.5
0.9917	290	6.5	1.5	5.5
0.9959	295	3.5	5.5	6
0.9923	300	3	6	5
0.9974	305	2.5	6.5	3
0.9885	310	0.5	6.5	3
0.9964	315	4.5	5.5	4.5
0.9788	320	0.5	3	7.5
0.9986	325	3	3.5	7
1.0039	330	2.5	5.5	4.5
0.9954	335	0.5	6	2.5
0.9999	340	1	5.5	4
0.9985	345	0.5	5.5	3.5
0.9956	350	2.5	5	4
0.9872	355	3.5	4	4.5
1.0122	360	1.5	4.5	4.5

**Table 3:** Comparison of measured and simulated values with and without proposed prototype.

Frequency (GHz)	Simulated, Directivity Only Horn (dBi)	Simulated, Gain Only Horn (dBi)	Measured, Directivity Only Horn (dBi)	Measured, Gain Only Horn (dBi)	Simulated, Directivity with Prototype (dBi)	Simulated, Gain with Prototype (dBi)	Measured, Directivity with Prototype (dBi)	Measured, Gain with Prototype (dBi)
10	18.886	18.841	18.900	18.945	20.500	19.700	21.168	20.869
10.5	19.458	19.430	19.500	19.528	21.800	21.100	20.978	20.532
11	19.698	19.702	19.600	19.596	22.000	21.600	23.109	22.942
11.5	20.028	19.994	20.100	20.134	21.700	21.400	22.393	22.128
12	20.489	20.463	20.310	20.335	22.100	21.900	23.372	23.007
12.5	20.687	20.673	20.500	20.514	22.600	22.300	23.505	23.151
13	21.025	20.976	21.010	21.060	22.500	22.300	22.839	22.645
13.5	21.255	21.228	21.100	21.128	23.000	22.800	23.87	23.305
14	21.480	21.452	21.400	21.428	23.400	23.000	23.875	23.432
14.5	21.759	21.721	21.800	21.838	23.200	22.600	23.319	22.795
15	21.956	21.927	21.900	21.930	22.200	21.200	23.558	22.524

**Table 4:** Comparison of proposed WBP structure against the other design prototypes.

References	Operating Band (GHz)	Electrical Area of Proposed Surface (mm)	Electrical Height from Feed Aperture (mm)	Lowest Operating Frequency (GHz)	Operating Frequency (GHz)	Peak Gain (dBi)	Peak Directivity (dBi)	Bandwidth (%)	3 dB Bandwidth (%)	Side Lobe Level (H-Plane) (dB)	Side Lobe Level (E-Plane) (dB)	Polarization	DBP/A	Thickness of Superstrate (mm)	Aperture Size	Fabrication Technique
Proposed	10 to 15	4.2 λ × 4.2 λ (105 × 105)	0.5 λ (12.5)	10	12	23.4	23.8	50	20	−15.5	−17.5	Linear	680	1.5 λ (37.5)	4.2 λ × 4.2 λ × 1.5 λ	Vero CMYK
[10]	11.2 to 12.8 (λ = 25 mm)	2.4 λ × 1.8 λ (60 × 60)	2.56 λ (63.9)	11.2	12	20	n/a	14.29	6.67	n/a	n/a	Linear	n/a	0.12 λ (3)	2.4 λ × 1.8 λ × 5.36 λ	Metal ring Patches
[13]	9 to 11 (λ = 30 mm)	6.67 λ × 6.67 λ (200 × 200)	0.16 λ (5)	9	10	22.5	n/a	15	7.69	n/a	n/a	Linear	n/a	0.24 λ (6)	6.67 λ × 6.67 λ × 0.24 λ	Metal Patches
[14]	11.8 to 15 (λ = 24 mm)	40.34 λ${}^{2}$ (Radius = 86)	n/a	11.8	12.5	24.2	n/a	25.6	26	n/a	−18.5	Linear	n/a	0.08 λ (2)	40.34 λ${}^{2}$ × 0.08 λ	Metal Patches
[15]	9.7 to 12.45 (λ = 27.42 mm)	19.84 λ${}^{2}$ (Radius = 68.9)	n/a	9.7	10.94	20.46	20.9	25.14	25	−20	−12	n/a	156	0.36 λ (10)	19.84 λ${}^{2}$ × 0.36 λ	Abrasive Waterjet Cutting
[17]	9 to 15 (λ = 23.07 mm)	1.24 λ${}^{2}$ (Radius = 43.81)	n/a	9	13	19	n/a	46.16	25	−22	−19	n/a	n/a	5.99 λ (138.3)	λ${}^{2}$× 5.99 λ	Fused Deposition Modeling
[19]	10 to 18 (λ = 25 mm)	4 λ × 4 λ (100 × 100)	0.5 λ (12.5)	10	12	25	25.5	66.67	28.57	n/a	−16 to −40	Linear	1479	0.6 λ (15)	4 λ × 4 λ × 10.1 λ	Multijet 3D Printing

## Data Availability

Could be provided upon request.
